# Editorial: MSC-Derived Extracellular Vesicles and Secreted Factors as “Cell-Free” Therapeutic Alternatives in Regenerative Medicine

**DOI:** 10.3389/fbioe.2022.842128

**Published:** 2022-01-26

**Authors:** Enrico Ragni, Ornella Parolini, Antonietta Rosa Silini

**Affiliations:** ^1^ IRCCS Istituto Ortopedico Galeazzi, Laboratorio di Biotecnologie Applicate All’Ortopedia, Milano, Italy; ^2^ Department of Life Science and Public Health, Università Cattolica Del Sacro Cuore, Rome, Italy; ^3^ Fondazione Policlinico Universitario “Agostino Gemelli” IRCCS, Rome, Italy; ^4^ Centro di Ricerca E. Menni, Fondazione Poliambulanza- Istituto Ospedaliero, Brescia, Italy

**Keywords:** mesenchymal stromal (or stem) cells, extracellular vesicle (EV), secretome, biomaterials, therapeutics

In recent years, the concept of “regenerative medicine” has gained interest in the clinical scenario. Within this broad field, mesenchymal stromal cells (MSCs) have paved the way for their potential to stimulate reparative and anti-inflammatory mechanisms to restore healthy functions in damaged organs and tissues (Gomez-Salazar et al., 2020). All these features are ascribed to MSCs capacity to interact with the diseased environment and release soluble mediators, both free and conveyed within extracellular vesicles (EVs) (Phinney and Pittenger, 2017), collectively termed the “secretome”. For these reasons, one of MSCs “fathers”, Prof. Arnold Caplan, in 2011 defined MSCs as an “injury drugstore” (Caplan and Correa, 2011) and recently proposed a nomenclature change in “medicinal signaling cells” (Caplan, 2017). Accordingly, MSC secretomes have repeatedly shown both *in vitro* and *in vivo* to maintain several features of the cells from which they derive, and are increasingly been used for both regenerative and immune modulatory applications (Harman et al., 2021).

Due to these reasons, the secretome itself or its constituents (cytokines/chemokines, EVs and embedded molecules) were assumed to serve as a cell-free option in the field of regenerative medicine (Pokrovskaya et al., 2020), avoiding controversial issues related to conventional cell-based therapies (Lukomska et al., 2019). In this frame, several protocols have been developed for MSC-EVs production under good manufacturing practice (Wei et al., 2021), and many clinical trials are currently completed or recruiting (Maumus et al., 2020). Nevertheless, several critical issues have been put aside in the excitement to pursue clinical trials. In particular, a thorough dissection of EVs potential, starting from their rigorous characterization to *in vitro/in vivo* evidence, is sometimes missing or underestimated. These deficiencies and pitfalls greatly limit MSC-EVs translation into clinical practice, ultimately dampening their therapeutic potential and opening the door to unsatisfactory effects.

This Research Topic presents cutting edge and innovative data on MSC secretome-based research, encompassing both innovative and, when possible, clinical production scalable characterization methods and new results in different disease settings, to select the most characterized and data-driven MSC-based cell free product.

The first step to frame the MSC secretome, and in particular EVs, is a thorough and reliable definition of techniques and parameters with the choice of the most appropriate isolation procedure being a crucial issue. To this end, Phan et al. proposed several methods to effectively and uniformly isolate MSC-EVs from complex biological milieu. By using multiple techniques including optical, non-optical and high-resolution single vesicle characterization methods, the authors clearly showed that isolation techniques greatly influence EVs composition and biological properties. In particular, not a single but multiple and complimentary techniques are crucial to reliably evaluate EVs potential on a more complex level. In this frame, the need of reliable albeit easy to manage protocols is mandatory. Ragni et al. showed how the molecular characterization of EVs, especially for miRNAs, is largely dependent on the selection of the most appropriate reference genes (RGs) since, to date, a universal RG miRNA for MSC-EVs is missing. The authors presented several RG miRNAs for adipose (adipose derived-MSC, ASC)- and bone marrow (bone marrow derived-MSC, BMSC)-derived EVs and, most importantly, showed how an integrated analysis encompassing different EV types is a mandatory step. Finally, Carlomagno et al. exhibited an innovative approach to characterize both EVs and whole secretome based on Raman spectroscopy, which is a non-destructive vibrational investigation method. Their results clearly emphasize how this technique can confirm EVs and secretome isolation reproducibility, a major issue for clinical translation when batch to batch differences depend not only on the source but also on the technical pipeline. These new methodologies will help in accelerating translation to the clinic and regulatory approval reducing concerns related to technical workflow.

The second step for the dissection of the biological role of secretome and EVs requires data on *in vitro* and preclinical efficacy, allowing *in vitro*–*in vivo* correlation of therapeutic potency. When data are largely missing, *in vitro* analyses are crucial. Ghebes et al. showed how BMSCs ability to support hematopoietic stem cells (HSPCs) expansion relies on EV-shuttled molecules, both proteins and miRNAs, and how the divergent molecular fingerprints in EVs from foetal or adult BMSCs may differentially support *ex vivo* HSPCs expansion. The issue of molecular signature and composition was also addressed by Zhong et al. in the frame of cardiovascular diseases and heart failure. In an *in vitro* model of doxorubicin-induced heart failure, miR-100-5p embedded in umbilical cord MSC-EVs inhibited oxidative stress, ROS and apoptosis increase through the direct downregulation of NOX4 expression. A similar role for embedded molecules was shown by Pan et al., who demonstrated the role of lncRNA malat-1 in MSCs-EVs to promote chondrocyte proliferation and repair as well as alleviate cartilage inflammation and degeneration *in vitro* and *in vivo* in osteoarthritis models. Consistently, in Qi et al., the use of an animal model allowed to sharply define the modulation of secretome given by the pre-activation of MSCs with serum from colitis rats, which promoted the production of paracrine factors improving the therapeutic effects on the disease. Of note, when data cover a number of different pathologies and indications, a more defined picture may be framed as reported from Cargnoni et al. and Sandonà et al., In the first manuscript related to inflammatory pathologies, perinatal derived EVs were described to directly modulate the immune response by targeting the inflammatory microenvironment. In the second work related to musculoskeletal diseases, MSCs and their secretome were reported to enhance both myofiber regeneration and myogenesis, with EVs postulated as candidates for cell free-based muscle regeneration. In this frame, EVs targeted delivery to the injured site or diseased tissue is another crucial need. To improve the process, Driscoll et al. fabricated an engineered biomaterial composed of EVs cross-linked with graphene oxide and exhibiting cell-type dependent cytotoxicity on liver cancer cells with minimal impact on healthy hepatocytes. Thus, next step in EVs production will be their manipulation under tissue engineering approaches for regenerative medicine.

Altogether, this Research Topic highlights the need for proper EVs characterization and functional studies, both mandatory prerequisites for a faster and bioengineering-driven EVs translation from bench to bedside.
